# The effects of assessment intensity on participant burden, compliance, within-person variance, and within-person relationships in ambulatory assessment

**DOI:** 10.3758/s13428-021-01683-6

**Published:** 2021-09-10

**Authors:** Kilian Hasselhorn, Charlotte Ottenstein, Tanja Lischetzke

**Affiliations:** Department of Psychology, University of Koblenz-Landau, Fortstr. 7, 76829 Landau, Germany

**Keywords:** Ambulatory assessment, Sampling frequency, Questionnaire length, Perceived burden, Compliance, Within-person variability, Within-person relationships

## Abstract

Considering the very large number of studies that have applied ambulatory assessment (AA) in the last decade across diverse fields of research, knowledge about the effects that these design choices have on participants’ perceived burden, data quantity (i.e., compliance with the AA protocol), and data quality (e.g., within-person relationships between time-varying variables) is surprisingly restricted. The aim of the current research was to experimentally manipulate aspects of an AA study’s assessment intensity—sampling frequency (Study 1) and questionnaire length (Study 2)—and to investigate their impact on perceived burden, compliance, within-person variability, and within-person relationships between time-varying variables. In Study 1, students (*n* = 313) received either 3 or 9 questionnaires per day for the first 7 days of the study. In Study 2, students (*n* = 282) received either a 33- or 82-item questionnaire three times a day for 14 days. Within-person variability and within-person relationships were investigated with respect to momentary pleasant-unpleasant mood and state extraversion. The results of Study 1 showed that a higher sampling frequency increased perceived burden but did not affect the other aspects we investigated. In Study 2, longer questionnaire length did not affect perceived burden or compliance but yielded a smaller degree of within-person variability in momentary mood (but not in state extraversion) and a smaller within-person relationship between state extraversion and mood. Differences between Studies 1 and 2 with respect to the type of manipulation of assessment intensity are discussed.

A growing body of literature is using ambulatory assessment (AA) in the fields of psychology and life science (Hamaker & Wichers, 2017). AA (also called daily diary, experience sampling, or ecological momentary assessment) is a method for assessing daily life experiences, for example, the ongoing behavior, experience, physiology, and environmental aspects of people in naturalistic and unconstrained settings (Fahrenberg, 2006). One of the main advantages of AA is that it allows researchers to study within-person dynamics (e.g., within-person relationships between time-varying variables) as well as individual differences in these within-person dynamics (Hamaker & Wichers, 2017). Furthermore, AA provides reduced recall bias and high ecological validity (Mehl & Conner, 2014; Trull & Ebner-Priemer, 2014).

When researchers plan an AA study, they have to make decisions about multiple design features in order to strike a balance between being able to gather rich information and ensuring that they do not overburden their participants (Carpenter et al., 2016). Some of these features consist of the types of reports to include (e.g., time-based, event-triggered), the number of days to survey people, the number of assessments to administer per day (sampling frequency), and the number of items to administer per measurement occasion (questionnaire length), but many other design features could also be considered (e.g., the population of interest, item content, item difficulty, item order, the instructions given to the participants, the software used to signal the participants, financial compensation). For more detailed information about study design considerations and methods of data collection, see, for example, Mehl and Conner (2014). In the present research, we focused on sampling frequency and questionnaire length as important aspects of assessment intensity.

Considering the very large number of studies that have applied AA in the last decade across diverse fields of research, knowledge about the effects that these design choices have on participants’ perceived burden, data quantity (i.e., compliance with the AA protocol), and data quality (e.g., careless responding or psychometric properties of measures) is surprisingly restricted (cf. Eisele et al., 2020). The (relatively scarce) previous methodological research on the effects of design-related characteristics on aspects of AA data (e.g., data quantity and data quality) can be divided into two groups: On the one hand, meta-analytic research has analyzed whether between-study differences in assessment intensity are related to between-study differences in compliance (e.g., Jones et al., 2019; Ottenstein & Werner, 2021; Vachon et al., 2019) and in the proportion of within-person variance in time-varying constructs (Podsakoff et al., 2019)—the latter being a characteristic of AA data that should be of particular interest to researchers who want to investigate within-person dynamics (Hamaker & Wichers, 2017). On the other hand, experimental research has manipulated assessment intensity in an AA study and analyzed its effects on participant burden (e.g., Eisele et al., 2020; Stone et al., 2003), compliance (e.g., Conner & Reid, 2012; Stone et al., 2003), and careless responding (Eisele et al., 2020). Whereas third variables cannot be ruled out in correlational analyses of between-study differences, experimental AA studies have the advantage that the internal validity of causal conclusions is higher. In the present research, we aimed to contribute to the literature in the following ways: First, with respect to assessment intensity as the independent variable, in Study 1, we manipulated sampling frequency to allow comparisons with previous research, whereas in Study 2, we manipulated questionnaire length, which (to our knowledge) has been experimentally manipulated in only one study (Eisele et al., 2020) to date. Second, with respect to characteristics of the AA data as dependent variables, our aim was to investigate both previously studied variables (perceived burden, compliance) and understudied variables (within-person variability, within-person relationships between time-varying variables). See Table 1 for an overview of and additional information (study design, studied design features, dependent variables, and results) about previous research in this area. In the following, we address each of these dependent variables in turn to derive our hypotheses (which were preregistered on the OSF, view-only link for review: https://osf.io/vw3gf/?view_only=b6f9f08a6b5941eb9c17a4951d1d0cd2).

**Table 1:** Previous studies on the effects of design features of AA studies on burden, compliance, within-person variability, and within-person relations between variables

Article	Study design	Pop.	Design feature (factor levels or range)	DVs	Results for (higher) SF	Results for (higher) QL
Conner and Reid (2012)	Exp.AA	NC	SF (1 vs. 3 vs. 6 qu./day)	Com	No effect on Com	—
Eisele et al. (2020)	Exp. AA	NC	SF (3 vs. 6 vs. 9 qu./day), QL (30 vs. 60 items)	Bur, Com	No effect on Bur, no effect on Com	Increase of Bur
Jones et al. (2019)	Meta/pooled	C	SF (1–9 qu./day)	Com	No effect on Com	—
McCarthy et al. (2015)	Exp. AA	NC	SF (1 vs. 6 qu./day)	Com	No effect on Com	—
Morren et al. (2009)	Meta/pooled	C, NC	SF (1–10 qu./day) QL (1–63 items)	Com	No effect on Com	Related to lower Com
Ono et al. (2019)	Meta/pooled	C	SF (3–12 qu./day), QL (6–63 items)	Com	No effect on Com	No effect on Com
Ottenstein and Werner (2021)	Meta/pooled	C, NC	SF (0.14–44 qu./day), QL (1–150 items)	Com	No effect on Com	No effect on Com
Podsakoff et al. (2019)	Meta/pooled	NC	—	WPV	Related to larger WPV	—
Rintala et al. (2019)	Meta/pooled	C, NC	SF (10 qu./day), QL (42–52 items)	Com	—	No effect on Com
Soyster et al. (2019)	Meta/pooled	C	SF (4 or 8 qu./day), QL (16–40 items)	Com	—	No effect on Com
Stone et al. (2003)	Exp. AA	C	SF (3 vs. 6 vs. 12 qu./day)	Bur, Com	Increase of Bur , no effect onCom	—
Vachon et al. (2019)	Meta/pooled	C	—	Com	Related to lower Com	No effect on Com
Walsh and Brinker (2016)	Exp. AA	NC	SF (20 items across 1 or 2 days)	Com	No effect on Com	—

*Note*. Pop = Population under study; DV = Dependent variable(s); Exp. AA = Experimental AA study; Meta/pooled = Meta-analysis or pooled data analysis; C = Clinical sample; NC = Nonclinical sample; SF = Sampling frequency; QL = Questionnaire length; qu = Questionnaire; Bur = Burden; Com = Compliance; WPV = Within-person variability.

## Perceived burden

For participants in an AA study, a higher (vs. lower) assessment intensity (e.g., more questionnaires per day or more items per questionnaire) means that participants have to invest more time and energy in participating in the study if they aim to be thorough (Santangelo et al., 2013). A higher perceived burden has been conceptualized as a process that can result in a reduction in the quantity and quality of AA data (Eisele et al., 2020; Fuller-Tyszkiewicz et al., 2013; Santangelo et al., 2013). Although several researchers have stated that increases in sampling frequency or questionnaire length increase perceived burden (Moskowitz et al., 2009; Napa Scollon et al., 2009; Santangelo et al., 2013), there are only a few empirical studies that have experimentally manipulated assessment intensity and analyzed perceived burden as an outcome (Eisele et al., 2020; Stone et al., 2003). In the study by Stone et al. (2003), participants with pain syndromes were randomly assigned to either no AA phase or sampling densities of 3, 6, and 12 questionnaires per day over 2 weeks. Perceived burden, which was assessed retrospectively after the AA phase, was higher in groups with a higher sampling frequency. In a sample of students, Eisele et al. (2020) analyzed the effects of sampling frequency and questionnaire length on perceived burden over 2 weeks. They operationalized perceived burden as momentary perceived burden, which was measured with each questionnaire, and as retrospective perceived burden, which was measured after the AA phase. Their results revealed no increase in perceived burden with a higher sampling frequency, but perceived burden did increase with longer questionnaires (Eisele et al., 2020). One reason for the difference in results between these experimental AA studies with respect to the effect of sampling frequency on perceived burden might be the population of interest (clinical vs. nonclinical). In the present research, we decided to target a nonclinical population (students), as Eisele et al. (2020) did. Moreover, given that Eisele et al.’s study is the only previous study that experimentally manipulated questionnaire length, and given that we know of no correlational papers or meta-analyses on this topic, more experimental research on the effect of this aspect of assessment intensity on perceived burden is needed.

## Compliance

In AA studies, high compliance rates per person are considered particularly important for obtaining a representative picture of individuals’ everyday experiences and behaviors (Stone et al., 2003) because missing data can lead to biased inferences about person-level (aggregated) data (Courvoisier et al., 2012). A higher (vs. a lower) sampling frequency can be assumed to potentially compromise compliance. In the case of more frequent or longer questionnaires, participants might try to intentionally reduce the burden by not responding to prompts or by completing only a portion of the items on a particular measurement occasion (Vachon et al., 2019).

Surprisingly, previous experimental AA studies that manipulated sampling frequency in clinical samples (Stone et al., 2003; 3 vs. 6 vs. 12 questionnaires per day for 14 days) or in nonclinical samples (Conner & Reid, 2012; 1, 3, or 6 daily questionnaires for 13 days, McCarthy et al., 2015; 1 vs. 6 daily questionnaires for 14 days; Eisele et al., 2020; 3 vs. 6 vs. 9 questionnaires for 14 days; Walsh & Brinker, 2016; 20 questionnaires for either 1 or 2 days) found that the experimental groups did not differ in their compliance. Meta-analyses and pooled data analyses have provided a somewhat mixed picture: Some studies found no support for the idea that a higher sampling frequency is related to lower compliance rates in clinical samples (Jones et al., 2019; Ono et al., 2019; Soyster et al., 2019) or in other (clinical samples mixed with nonclinical) samples (Morren et al., 2009), whereas a recent meta-analysis that focused on AA studies in mental health research (Vachon et al., 2019) found lower compliance rates in studies that administered larger numbers of questionnaires per day.

With respect to questionnaire length, Eisele et al. (2020) found that longer questionnaires (60 items) led to lower compliance than shorter questionnaires (30 items). To our knowledge, this study is the only one to ever experimentally manipulate questionnaire length between conditions. Most meta-analyses and pooled data analyses have found no support for the idea that a longer questionnaire leads to lower compliance (Jones et al., 2019; Ono et al., 2019; Rintala et al., 2019; Soyster et al., 2019; Vachon et al., 2019) with the exception of Morren et al. (2009), who found that compliance was positively associated with shorter questionnaires.

Taken together, given that evidence of an effect of sampling frequency on compliance has been mixed, there is a need to scrutinize this effect further. Also, given that only one previous study manipulated questionnaire length (Eisele et al., 2020), there is also a need for more experimental research on the effect of questionnaire length on compliance.

## Within-person variability

Within-person variability is a prerequisite for investigating within-person dynamics (Heck & Thomas, 2015; Hox, 2002; Raudenbush & Bryk, 2002), and researchers have warned that research on within-person relationships between time-varying variables should not be conducted when within-person variability is low (Podsakoff et al., 2019; Rosen et al., 2016; Sonnentag et al., 2008; Trougakos et al., 2008).

In AA studies with a higher sampling frequency or longer questionnaire length, participants might become more fatigued over time (e.g., Beal, 2015) and might consequently respond in a more heuristic, less nuanced way to repeated prompts, thereby reducing the degree of within-person variability in time-varying variables (Fuller-Tyszkiewicz et al., 2013; Podsakoff et al., 2019). With respect to sampling frequency, Podsakoff et al. (2019) added that another process might work in the opposite direction: More frequent prompts might give participants the opportunity to become more aware of differences between a current state and previous states, thereby potentially increasing within-person variability in time-varying variables.

Empirical evidence on whether higher (vs. lower) assessment intensity has an effect on the degree of within-person variability in time-varying variables is scarce. Two AA studies that analyzed whether the degree of within-person variability changed over the course of an AA study have provided indirect evidence: In a community sample of young women, Fuller-Tyszkiewicz et al. (2013) found that the within-person variability in state body dissatisfaction scores declined as a function of the number of days into the study when they were measured. In a sample of depressed patients, Vachon et al. (2016) analyzed the trajectory of within-person variability in psychological states across a twice-daily AA study that spanned a total of 5 months. Their results revealed a decrease in the variability of cognitive (e.g., rumination) and affective (e.g., depressed mood) states across the course of the study. Podsakoff et al. (2019) conducted a meta-analysis on AA studies that had obtained their data from working employees and analyzed whether between-study differences in sampling frequency (number of questionnaires per day) and study duration (number of days) were related to between-study differences in the proportion of within-person variance. Among the time-varying constructs that were included, momentary affect and stressors were studied the most. Podsakoff et al. (2019) found that higher sampling frequency (but not study duration) predicted larger within-person variability. Taken together, the findings of these studies provide a mixed picture. Moreover, the evidence is not fully conclusive for methodological reasons: When analyzing within-person variability as a function of the amount of time into the study, variables that were confounded with the day of the week (e.g., change in compliance on weekdays compared with weekend days; Phillips et al., 2014), the day (e.g., changes in daily activities), or the month (e.g., seasonal effects) might provide alternative explanations. Likewise, in correlational analyses at the level of studies, between-study differences in third variables could have driven the effect. Hence, in the present research, we followed Podsakoff et al.’s (2019) suggestion that “scholars may find experimental designs to be particularly well suited to addressing research questions about the effect of study design on variance in measures” (p. 15). To our knowledge, no study has yet analyzed the effect of manipulations of assessment intensity on within-person variability.

## Within-person relationships between time-varying variables

A great deal of research on within-person dynamics tends to focus on within-person relationships between time-varying variables (Liu et al., 2019; May et al., 2018; Sitzmann & Yeo, 2013). Fuller-Tyszkiewicz et al. (2013) argued that a decrease in within-person variability can lead to a decline in the strength of within-person relationships between time-varying variables. Empirically, however, the decrease in within-person variability that was found did not translate into smaller within-person relationships as a function of the number of days into the study (Fuller-Tyszkiewicz et al., 2013).

To our knowledge, no AA studies to date have investigated the effect of experimentally manipulated assessment intensity on within-person relationships between time-varying variables. Therefore, more experimental research on the effect of assessment intensity on within-person relationships between time-varying variables is needed.

## The current research

The aim of the current research was to experimentally manipulate sampling frequency (Study 1) and questionnaire length (Study 2) as aspects of an AA study’s assessment intensity and to investigate their impact on perceived burden, compliance, within-person variability, and within-person relationships between time-varying variables. We expected that higher assessment intensity would increase perceived burden (Hypothesis 1) and would decrease compliance (H2), within-person variability (H3), and within-person relationships between time-varying variables (H4). Note that we preregistered these hypotheses in February 2019 (Study 1) and in January 2020 (Study 2) and that some of the previous research we cited had not been published at that time.^1^

To test Hypothesis 1 on perceived burden, we decided to assess perceived burden as both a daily and a retrospective burden, similar to Eisele et al.’s (2020) study. To test Hypothesis 3 on within-person variability and Hypothesis 4 on within-person relationships, we selected momentary mood and state extraversion as two time-varying constructs that (a) have frequently been assessed in previous research, (b) have been shown to possess adequate within-person variability (e.g., McCabe & Fleeson, 2016; Podsakoff et al., 2019), and (c) have been shown to be related within persons across multiple studies (e.g., Fleeson et al., 2002; Lischetzke et al., 2012; McNiel et al., 2010; McNiel & Fleeson, 2006).

## Study 1

Previous AA studies on state personality have typically administered four to five questionnaires per day (e.g., Fleeson & Gallagher, 2009). AA studies on momentary mood have shown a larger range of sampling frequencies: Studies assessing mood in the field of applied psychology have typically included one to three questionnaires per day (Podsakoff et al., 2019), whereas studies in the field of affect dynamics research have typically included seven to 10 questionnaires per day (Dejonckheere et al., 2019). For our experimental manipulation of sampling frequency in Study 1, we therefore selected a low and a high number within this observed range (three vs. nine questionnaires per day).

## Method

### Study design

The study consisted of an initial online survey, an AA phase across 7 days with either three or nine questionnaires per day (depending on the experimental condition that participants had been randomly assigned to), and a retrospective online survey. For the AA phase, participants chose a specific time schedule that best fit their waking hours (9:00–21:00 or 10:30–22:30). In the low sampling frequency group, the three questionnaires per day were distributed evenly across the day. In the high sampling frequency group, the first, the fifth, and the ninth questionnaire were scheduled at the same time of day as the three questionnaires from the low sampling frequency group, and the six additional questionnaires were distributed between these questionnaires (see Table 2 for more detailed information).

**Table 2:** Sampling scheme of Study 1

Time of day	Experimental group	
	Low sampling frequency	High sampling frequency
9:00–10:40	Questionnaire 1	Questionnaire 1
11:00–13:50		Questionnaires 2–4
14:10–15:50	Questionnaire 2	Questionnaire 5
16:10–19:00		Questionnaires 6–8
19:20–21:00	Questionnaire 3	Questionnaire 9

*Note*. The displayed sampling scheme refers to the first of the two time schedules from which participants could choose (9:00–21:00 vs. 10:30–22:30). That is, in the second time schedule, each questionnaire was scheduled 90 min later. For each questionnaire, participants had the option to delay their response for up to 15 min. In the high sampling frequency group, Questionnaires 2, 3, and 4 and Questionnaires 6, 7, and 8 were at least 28 min apart.

After the 7-day AA phase, a second 7-day AA phase followed immediately. This time, the sampling frequency was switched between the groups. This was done to ensure that each participant invested a comparable amount of time participating in the study so that the financial compensation, which was the same for both groups, was fair. Given that our focus was on the between-group comparison (high vs. low sampling frequency), and not on the effect of switching sampling frequency within persons, the analyses in the present paper are based on the data from the first 7-day AA phase.

During the initial online survey, participants completed a demographic questionnaire and trait self-report measures. In each AA questionnaire, participants rated their momentary motivation, time pressure, mood, clarity of mood, and state personality (extraversion and conscientiousness). In the last AA questionnaire each day, participants additionally rated their daily stress and perceived burden. In the retrospective online survey, participants rated their perceived burden and careless responding with respect to the past 7 days (as well as other constructs that were not relevant for the present analyses: retrospective mood, clarity of mood, and attention to feelings).

### Participants

Participants were required to be currently enrolled as a student and to be in possession of an Android smartphone. Participants were recruited via flyers, posters, e-mails, and posts on Facebook during students’ semester breaks.

As most hypotheses in this study focused on group differences, we based our sample size considerations on the power to detect a small to moderate (*d* = 0.30) mean difference (independent-samples *t* test). We needed 278 participants to achieve a power of .80.

A total of 474 individuals filled out the initial online survey. Due to technical problems with the software for the AA phase, various participants could not synchronize their smartphone with our study and withdrew their participation. One of the reasons for dropout was that participants with an iOS smartphone realized only at this stage that participation required an Android smartphone, as had been indicated in the information we gave them about the study. A total of 318 individuals took part in the AA phase that followed (155 individuals in the low sampling frequency condition), and 200 individuals responded in time to the retrospective online survey after the first 7 days (within the prespecified time frame of 12 hr). Participants who did not respond to the retrospective online survey were not excluded from the data analyses. Data from five participants were excluded from the analyses due to careless responding (see the data cleaning section). The final sample consisted of 313 students (low sampling frequency group: 86% women; age range: 18 to 34 years, M = 23.18, SD = 3.23; high sampling frequency group: 83% women; age range: 18 to 40 years, M = 23.98, SD = 4.12).

### Procedure

All study procedures were approved by the psychological Ethics Committee at the University Koblenz-Landau, Germany. After informed consent was obtained, the study began with the initial online survey. Subsequently, participants were randomly assigned to one of two experimental conditions (low sampling frequency or high sampling frequency) and randomly assigned to a starting day of the week. Prior to their AA phase, participants received a manual that explained how to install and run the smartphone application movisensXS, Versions 1.4.5, 1.4.6, 1.4.8, 1.5.0, and 1.5.1 (movisens GmbH, Karlsruhe, Germany) and connect to our study as this was required for participation. Participants were told that the number of questionnaires administered per day would range from three to nine times over the 2 weeks. At each measurement occasion, participants could either respond to the questionnaire or delay their response for up to 15 min. Participants who missed the first alarm were signaled 5 min later. If the questionnaire was not started by 15 min after the first signal, the questionnaire became unavailable. At the end of the 7th day of each AA phase (21:00 for participants with the early time schedule or 22:30 for participants with the later time schedule), participants were sent a link to the retrospective online survey via e-mail. This online survey had to be completed within a 12-hr time frame. Students were given 15€ in exchange for their participation if they had answered at least 50% of the AA questionnaires and had the chance to win 25€ extra if they had answered at least 80% of the AA questionnaires. Furthermore, at the end of the second retrospective online survey, they could indicate whether they wished to receive personal feedback regarding the constructs measured in the study after their participation was complete. In the low sampling frequency group (high sampling frequency group), 98 (92) participants requested feedback, 10 (10) participants did not want feedback, and 45 (58) participants did not answer the item.

### Data cleaning

To screen for careless responding, we analyzed inconsistent responding across reverse-poled items (see below) and a “Use Me” item (Meade & Craig, 2012). First, data from five participants (three in the high sampling frequency group) who indicated in the retrospective online survey that their data should not be used in our analyses were excluded from the analyses. The remaining 313 participants had completed 9158 AA questionnaires.

Subsequently, we removed 332 AA questionnaires (149 AA questionnaires in the low sampling frequency group) due to inconsistent responding (Meade & Craig, 2012) across the reverse-poled (mood) items.^2^ Because these questionnaires had been completed by the participants, compliance was unaffected by the AA questionnaires that were removed (see the Measures section). Hence, our analyses were based on 8528 AA questionnaires nested in 313 participants (with the exception of the compliance analysis, which was based on 9158 AA questionnaires nested in 313 participants).

**Measures**^3^**.** ***Male.*** A factor was used to indicate gender, with a value of 0 for female participants and 1 for male participants.

### Feedback

A factor was used to indicate whether participants wanted feedback, with a value of 0 for participants who did not want to receive feedback and 1 for participants who wanted to receive feedback.

### *Sampling frequency*

A factor was used to indicate the sampling frequency, with a value of 0 for the low sampling frequency group and 1 for the high sampling frequency group.

### Momentary mood

We measured momentary (pleasant-unpleasant) mood with an adapted short version of the Multidimensional Mood Questionnaire (Steyer et al., 1997) that has been used in previous AA studies (Lischetzke et al., 2012; Ottenstein & Werner, 2021). Participants indicated how they *felt at the moment* on four items (bad-good [reverse-scored], unwell-well, unhappy-happy [reverse-scored], and unpleased-pleased). The response format was a seven-point Likert scale with each pole labeled (e.g., 1 = *very unwell* to 7 = *very well*). We calculated a mean score across the items so that a higher score indicated more pleasant mood. The within-person ω (Geldhof et al., 2014) was .91, and the between-person ω was .99.

### State extraversion

We measured state extraversion by taking the adjectives that McCabe and Fleeson (2016) had introduced for each subcomponent (sociability, assertiveness, and talkativeness) and modifying them so that they formed three bipolar items (one for each subcomponent). Participants indicated how they *behaved in the last half hour* on the items (outgoing-unsociable [reverse-scored], unassertive-assertive, and talkative-quiet [reverse-scored]. The response format was a seven-point Likert scale with each pole labeled (e.g., 1 = *very quiet* to 7 = *very talkative*) plus an extra category (i.e., *not applicable*) if the respondent wanted to skip the question (displayed below the Likert scale). We used a bipolar (instead of a unipolar) response format to have a common response format for the time-varying dependent variables (momentary mood, state extraversion). We calculated a mean score across items so that a higher score indicated more extraverted behavior. The within-person ω (Geldhof et al., 2014) was .80, and the between-person ω was .91.

### Daily perceived burden

Daily perceived burden was measured using three items from Stone et al. (2003). Participants were asked on a seven-point Likert scale (1 = *not at all* to 7 = *very much so*): “How much of a burden was it to participate in the study during the day?” “How much did participating in the study interfere with your usual activities?” and “How much were you annoyed with the number of times you were signaled per day?” We calculated a mean score across items so that a higher score indicated more perceived burden. The within-person ω (Geldhof et al., 2014) was .78, and the between-person ω was .94.

### Retrospectively perceived burden

Retrospectively perceived burden was measured with the same three items as daily perceived burden with the modification that they referred to the previous seven-day AA phase. We calculated a mean score across items so that a higher score indicated more retrospectively perceived burden. Revelle’s omega total (McNeish, 2018) was .79.

### Compliance

Compliance at the questionnaire level was defined as having responded to the last item on the AA questionnaire (coded 1 = *yes* and 0 = *no*). We calculated the relative compliance across all questionnaires for each person so that a higher score indicated more compliance.

### Data analytic methods

Hypothesis 1 (on the between-group difference in perceived burden) and Hypothesis 2 (on the between-group difference in compliance) were tested with two-level regression models with daily perceived burden (and daily compliance) at Level 1 and persons at Level 2. To test group differences in the respective variables, we included sampling frequency at the person level.^4^ Effects on retrospective perceived burden (Hypothesis 1) were tested with an independent-samples *t* test using R. Afterwards, we corrected for multiple testing using Benjamini and Hochberg’s (1995) procedure for controlling the false discovery rate (FDR) in H1 and H3. When testing for differences between groups, we corrected for two multiple tests (daily and retrospective perceived burden). Hypothesis 3 (on the between-group difference in within-person variability) was analyzed using a multigroup multilevel model for questionnaires nested in persons in Mplus (Muthén & Muthén, 1998–2017). We applied the latent variable modeling procedure proposed by Dowling et al. (2018) to evaluate between-group differences in within-person variability. The model decomposes the total variance into between-person variance and within-person variance for each group. By using the Mplus MODEL CONSTRAINT option, the statistical significance of the between-group difference in within-person variance can be tested. To compare within-person variability between the experimental groups, within-person variance was estimated on the basis of the three surveys that were scheduled at the same times across groups (i.e., in the low sampling frequency group, the three daily surveys were used; and in the high sampling frequency group, the corresponding first, fifth, and ninth surveys of the day were used; see Table 2). Given that fluctuations in mood follow a diurnal rhythm (see, e.g., Thayer, 1978; Watson, 2000), using only the surveys that were scheduled at the same times across groups allowed us to rule out time of day as an alternative explanation for potential between-group differences in within-person variability. As in Hypothesis 1, we corrected for two multiple tests (momentary mood and state extraversion) by applying the procedure presented by Benjamini and Hochberg (1995). Hypothesis 4 (on between-group differences in within-person relationships) was analyzed with two-level regression models with questionnaires at Level 1 and persons at Level 2. Person-mean-centered state extraversion was used as a Level 1 predictor of momentary mood. Level 2 random intercepts and random slopes were included in the model (LeBeau et al., 2018). To test whether the two experimental conditions differed in their association between state extraversion and momentary mood, we additionally included the cross-level interaction between sampling frequency at the person level and state extraversion at the questionnaire level.

Additionally, as suggested by the reviewers during the peer review process, we conducted several exploratory analyses. First, we explored whether age, gender, and feedback at Level 2 would be found to predict compliance. Previous research has provided a mixed picture of the effects of age and gender on compliance (Ono et al., 2019; Rintala et al., 2019; Soyster et al., 2019; Vachon et al., 2019).

Second, we explored within-person differences in the effects of different sampling frequencies (which were switched after 7 days) on daily perceived burden. To do so, we tested a multilevel model including both weeks of the AA phase with a Week (at the questionnaire level) x Sampling Frequency (at the person level) cross-level interaction.

For Hypothesis 1, the *t* test was computed in the R environment (R Core Team, 2020). All multilevel regression models were computed with the R package lme4 (Bates et al., 2015), and the *p* values were created with the R package lmerTest (Kuznetsova et al., 2017). The procedure by Dowling et al. (2018) and the multilevel reliabilities were computed in Mplus 8.3 (Muthén & Muthén, 1998–2017).

## Results

To test our hypotheses, which involved a directional prediction, we employed one-sided significance tests (Cho & Abe, 2013). Accordingly, we interpreted one-tailed *p* values and corresponding two-sided 90% confidence intervals for these tests (i.e., for the difference between the group means). Some of the reported tests (e.g., intercorrelations among the study variables, the main effect of the experimental group on momentary mood in the multilevel model) did not refer to a directional prediction, and hence, we reported two-sided *p* values for these estimates. In the text, we explicitly labeled the *p* values as one-sided when this applied.

Table 3 presents the means, standard deviations, and within- and between-person correlations between the variables for each group separately. For all momentary and daily measures, there was a substantial amount of within-person variance, ranging from 56% for momentary mood to 79% for state extraversion for the low sampling frequency group and from 54% for momentary mood to 79% for state extraversion for the high sampling frequency group.

**Table 3:** Descriptive statistics and bivariate correlations for the main variables presented separately for each experimental group (Study 1)

Group	Variable	1	2	3	4
Low sampling frequency	1. Pleasant-unpleasant mood	—	.24***	– .03	
	2. State extraversion	.33***	—	.12**	
	3. Daily perceived burden	– .21**	– .12	—	
	4. Retrospective perceived burden	– .12	.05	.72***	—
	*M*	5.12	4.31	2.00	2.20
	*SD*_*within*_	0.92	1.42	0.72	-
	*SD*_*between*_	0.80	0.70	0.54	0.75
	*N*_*persons*_^*a*^	153	151	149	93
	*N*_*questionnaires*_^*b*^	2295	1794	788	
High sampling frequency	1. Pleasant-unpleasant mood	—	.28***	– .10**	
	2. State extraversion	.25**	—	.04	
	3. Daily perceived burden	– .31***	.13	—	
	4. Retrospective perceived burden	– .14	.16	.82***	—
	*M*	5.00	4.19	2.56	2.82
	*SD*_*within*_	0.87	1.40	0.71	-
	*SD*_*between*_	0.79	0.71	0.59	0.75
	*N*_*persons*_^*a*^	160	160	154	101
	*N*_*questionnaires*_^*b*^	2281	1769	791	

*Note*. Between-person correlations are presented below the diagonal. Within-person correlations between the daily measures are presented above the diagonal. All *p* values are two-sided *p* values. For all daily measures, we extracted the mean (intercept) and standard deviation from the multilevel null model of the respective variable.

^a^*N* differed between momentary mood and state extraversion because of the “*not applicable*” response option in state extraversion. ^b^*N* differed because the respective variables were assessed on different measurement occasions.

*p < .05. **p < .01. ***p < .001.

### Perceived burden

In line with Hypothesis 1, the low sampling frequency group (M = 2.01, SD = 0.64) perceived a lower daily burden than the high sampling frequency group (M = 2.56, SD = 0.70), *t*(291) = 7.47, one-tailed *p* < .001, 90% CI [0.44, 0.69], *d* = 0.83. This finding remained significant after we corrected the false discovery rate.

Similarly, for the retrospective measure, the low sampling frequency group (M = 2.20, SD = 0.75) perceived a significantly lower retrospective burden than the high sampling frequency group (M = 2.82, SD = 0.75), *t*(192) = 5.78, one-tailed *p* < .001, 90% CI [− 0.80, − 0.44], *d* = 0.83. This finding also remained significant after we corrected the false discovery rate.^5^

### Compliance

Contrary to Hypothesis 2, the low sampling frequency group (M = 0.71, SD = 0.25) did not demonstrate higher compliance than the high sampling frequency group (M = 0.68, SD = 0.25), *t*(311) = – 1.28, one-tailed *p* =.101, 90% CI [− 0.08, 0.01], *d* = − 0.15.

### Within-person variability

Contrary to Hypothesis 3, the low sampling frequency group (estimate = 0.83, SE = 0.06) did not show a significantly higher within-person variance in momentary mood than the high sampling frequency group (estimate = 0.75, SE = 0.05), *z* = − 1.20, one-tailed *p* = .116, 90% CI = [− 0.03, 0.21], *d* = − 0.10.^6^

Similarly, for state extraversion, the low sampling frequency group (estimate = 2.01, SE = 0.12) did not show a significantly higher within-person variance than the high sampling frequency group (estimate = 1.94, SE = 0.11), *z* = – 0.43, one-tailed *p* = .336, 90% CI = [– 0.20, 0.33], *d* = – 0.08.^3^

### Within-person relationships between time-varying variables

As can be seen in Table 4 (Model 2), unexpectedly, the cross-level interaction term (for the interaction between sampling frequency at Level 2 and state extraversion at Level 1) had a positive sign, which means that the low sampling frequency group (*b* = 0.16) had a descriptively smaller regression coefficient for state extraversion than the high sampling frequency group (*b* = 0.21). The cross-level interaction term was not significantly different from zero, *t*(198) = 1.35, one-tailed *p* = .911, 90% CI [− 0.01, 0.09].

**Table 4:** Multilevel model (fixed effects) predicting momentary mood by state extraversion and sampling frequency (Study 1)

Model Predictor	Estimate	*SE*	*df*	*t*
Model 1:				
Intercept	5.16			
State extraversion	0.18	0.02	199.2	11.84***
Sampling frequency	– 0.12	0.10	297.0	– 1.24
Model 2:				
Intercept	5.17			
State extraversion	0.16	0.02	193.6	7.43***
Sampling frequency	– 0.14	0.10	299.0	– 1.44
State Extraversion x Sampling Frequency	0.04	0.03	198.5	1.35

*Note*. State extraversion was centered at the person mean. Sampling frequency was coded as 0 = low sampling frequency group and 1= high sampling frequency group.

***p < .001.

### Exploratory data analysis. *Predictors of compliance*

None of the variables from the exploratory analyses were significantly related to compliance: gender, *t*(203) = 0.20, *p* = .840, 95% CI [− 0.07, 0.09]; age, *t*(202) = 0.72, *p* = .475, 95% CI [– 0.004, 0.010]; requesting personal feedback, *t*(204) = 0.12, *p* = .905, 95% CI [– 0.09, 0.10].

### Effects of sampling frequency as a within-person factor

In a multilevel model with sampling frequency (low vs. high) as a within-person factor, order (low sampling frequency first vs. high sampling frequency first) as a between-person factor, and their cross-level interaction, a main effect of sampling frequency (b = 0.94, SE = 0.06), t(269) = 16.36, *p* < .001, 95% CI [0.82, 1.05], and a main effect of order (b = – 0.38, SE = 0.07), t(282) = – 5.32, *p* < .001, 95% CI [– 0.52, – 0.24], emerged. The cross-level interaction term was not significantly different from zero (b = 0.02, SE = 0.08), t(270) = 0.25, *p* = .804, 95% CI [– 0.14, 0.18]. That is, during the high sampling frequency phase, participants reported a higher burden than during the low sampling frequency phase, and in the group that had started with the high sampling frequency phase, subjective burden values were lower, on average, than in the group that had started with the low sampling frequency phase.

### Discussion

Using an experimental manipulation of sampling frequency in an AA study, we found that, as expected, a higher sampling frequency led to higher perceived burden (H1). However, contrary to our expectations, the high versus low sampling frequency groups did not differ in compliance (H2), within-person variability in momentary mood and state extraversion (H3), or the within-person relationship between momentary mood and state extraversion (H4).

Our finding that the sampling frequency had an effect on perceived burden is in line with previous assumptions (Moskowitz et al., 2009; Santangelo et al., 2013) and with the empirical research by Stone et al. (2003). Contrary to our results, Eisele et al. (2020) found no effect of sampling frequency on perceived burden. A possible explanation could be that in Eisele et al.’s study, the effect of sampling frequency “was canceled out by the increased motivation due to the higher incentive” (Eisele et al., p. 12) in the high sampling frequency group (40 vs. 80 euros in the group with three vs. nine AA questionnaires per day, respectively), whereas in our study, financial compensation for the complete study did not differ between the experimental groups.

Our finding that the sampling frequency had no effect on compliance is in line with previous research (Conner & Reid, 2012; Eisele et al., 2020; McCarthy et al., 2015; Ono et al., 2019; Soyster et al., 2019; Stone et al., 2003; Walsh & Brinker, 2016). The results indicate that the higher burden that was reported in the high sampling frequency group did not translate into less effort in responding to the AA prompts. An ad hoc explanation is that participants in the high sampling frequency group might have kept up a similarly high compliance rate (despite perceiving a higher burden) because they wanted the personal feedback after study participation to show a representative picture of their experience and behavior during the study.

To our knowledge, this study is the first to analyze the effect of experimentally manipulated sampling frequency on within-person variability and the within-person relationship between time-varying variables. Although it might sound like good news to researchers applying AA designs to study within-person dynamics that we did not find differences between the low and the high sampling frequency groups, it seems premature to conclude that assessment intensity has no effect on within-person (co)variability. Hence, in Study 2, we aimed to conceptually replicate this finding by using a different manipulation of assessment intensity (questionnaire length).

## Study 2

In Study 2, we wanted to conceptually replicate and generalize our findings from Study 1 with a different manipulation of an aspect of assessment intensity. Therefore, we chose to manipulate the questionnaire length per questionnaire as another central design characteristic instead of the sampling frequency. Moreover, we extended the duration of the AA phase from 1 to 2 weeks.

Previous meta-analyses and pooled data analyses have included studies with different ranges of numbers of items (see Table 1). Our aim was to maximize the difference in questionnaire length between the groups (in a range that was realistic for AA studies) while holding constant the constructs that were measured across groups (by using short vs. long forms for each construct).

### Method

#### **Study design**

The study consisted of an initial online survey, an AA phase across 14 days with three short or long questionnaires per day (depending on the experimental condition that participants had been randomly assigned to), and a retrospective online survey.

The short questionnaire group had to answer 33 items (or 36 items in the evening) per questionnaire, and the long questionnaire group had to answer 82 items (or 85 items in the evening). The average response time for one questionnaire in the short questionnaire group (M = 1.65 min, SD = 0.63) was lower, on average, than in the long questionnaire group (M = 3.89 min, SD = 3.42). The two groups answered questions about the same constructs. This allowed us to investigate the effect of questionnaire length without the confounding effect of measuring different constructs between the groups. The difference in the number of items between these groups was achieved by using a short versus a long version of the measures of the constructs (see the Measures section).

During the initial online survey, participants completed a demographic questionnaire and trait self-report measures. In each AA questionnaire, participants rated their momentary motivation, time pressure, state personality, situation characteristics, and momentary mood. In the last AA questionnaire per day, participants additionally rated their perceived burden. In the retrospective online survey, participants rated their retrospective mood, perceived burden, and careless responding regarding the past 14 days. Additionally, participants rated their trait personality again.

#### Participants

Participants were required to be currently enrolled as a student, to be in possession of a smartphone, to speak German, and to be at least 18 years old. Participants were recruited via flyers, e-mails, and posts on Facebook in January, and the last questionnaire was sent to participants on February 10, 2020. The a priori power analysis was conducted in the same way as in Study 1.

A total of 303 individuals filled out the initial online survey, 284 individuals took part in the AA phase that followed (142 individuals in the short questionnaire condition), and 235 individuals responded to the retrospective online survey after the AA phase (within the prespecified time frame of 5 days). Participants who did not respond to the retrospective online survey were not excluded from the data analyses. Data from two participants were excluded from the analyses due to careless responding (see the Data Cleaning section). The final sample consisted of 282 students (short questionnaire group: 83% women; age range 18 to 39 years, M = 23.20, SD = 3.45; long questionnaire group: 87% women; age range 18 to 55 years, M = 22.90, SD = 3.81).

#### Procedure

All study procedures were approved by the psychological Ethics Committee at the University Koblenz-Landau, Germany. After obtaining informed consent, the study began with an initial online survey to assess trait measures and sociodemographic information. Subsequently, participants were randomly assigned to one of two experimental conditions (short questionnaire or long questionnaire) and were informed about the upcoming AA phase at least 2 days in advance. The AA phase of 14 days began on the next possible Monday or Thursday. All participants received three links to questionnaires via SMS per day (10:00, 14:00, and 18:00) and had 45 min until they could no longer start the questionnaire. After the 14-day AA phase, participants received a link to the retrospective online survey via SMS. This online survey had to be completed within a 5-day time frame. Participants received up to 30€ in exchange for their participation depending on their compliance rate (25% = 3€, 50% = 10€, 75% = 20€, and 90% = 30€). Furthermore, when they filled out the initial online survey, they could choose to receive personal feedback regarding the measured constructs after they participated. In the short questionnaire group (long questionnaire group) 133 (131) participants requested feedback, and 9 (9) participants did not want feedback.

#### Data cleaning

To screen for careless responding, we analyzed inconsistent responding across reverse-poled items (see below) and a “Use Me” item (Meade & Craig, 2012). First, data from two participants who indicated in the retrospective online survey that their data should not be used in the analyses were excluded from the analyses. The remaining 282 participants had completed 8611 AA questionnaires. Finally, we removed 26 AA questionnaires (14 AA questionnaires in the short questionnaire group) due to inconsistent responding (Meade & Craig, 2012) across reverse-poled (mood) items.^7^ Because these questionnaires had been completed by the participants, compliance was unaffected by the AA questionnaires that were removed (see the Measures section). Hence, our analyses were based on 8585 AA questionnaires nested in 282 participants (with the exception of the compliance analysis, which was based on 8611 AA questionnaires nested in 282 participants).

#### Measures^8^

The constructs that were measured with fewer items in the short questionnaire group compared with the long questionnaire group were situation characteristics (8 vs. 32 items), pleasant-unpleasant mood (2 vs. 4 items), calm-tense mood (1 vs. 2 items), wakefulness-tiredness (1 vs. 2 items), and state openness to experience, agreeableness, and neuroticism (1 vs. 8 items). As a result, we achieved variation in questionnaire length while at the same time measuring the same constructs in the two groups. Only the items that were included in the short questionnaire group (which were the ones analyzed in both groups) will be described in the following (for the additional items assessed in the long questionnaire group, see the supplemental online material).

#### Male

A factor was used to indicate gender, with a value of 0 for female participants and 1 for male participants.

#### Feedback

A factor was used to indicate whether participants wanted feedback, with a value of 1 for participants who wanted to receive feedback and 0 for participants who did not want to receive feedback.

#### Questionnaire length

A factor was used to indicate questionnaire length, with a value of 0 for the short questionnaire and 1 for the long questionnaire.

#### Momentary mood

To measure momentary (pleasant-unpleasant) mood, we used two items from Study 1 (bad-good [reverse-scored], unwell-well). We calculated a mean score across two mood items so that a higher score indicated more pleasant mood. The within-person α (Geldhof et al., 2014) was .86, and the between-person α was .97.

#### State extraversion

We measured state extraversion with an adapted version of the adjectives from Saucier’s (1994) unipolar Big Five Mini-Markers (Comensoli & MacCann, 2015). Participants indicated how they *behaved in the last half hour* on eight items (bashful [reverse-scored], bold, energetic, extraverted, quiet [reverse-scored], shy [reverse-scored], talkative, and withdrawn [reverse-scored]). The response format was a five-point Likert scale with each pole labeled (1 = *extremely inaccurate* to 5 = *extremely accurate*). We calculated a mean score across the eight items so that a higher score indicated more extraverted behavior. The within-person ω (Geldhof et al., 2014) was .72, and the between-person ω was .59.

#### Daily *p*erceived burden

To measure daily perceived burden, we used the same items as in Study 1. We calculated a mean score across items so that a higher score indicated more perceived burden. The within-person ω (Geldhof et al., 2014) was .71, and the between-person ω was .91.

#### Retrospective perceived burden

Retrospectively perceived burden was measured with the same three items as in Study 1. Revelle’s omega total (McNeish, 2018) was .82.

#### Compliance

Compliance at the questionnaire level was defined as having responded to the last item on the AA questionnaire (coded 1 = *yes* and 0 = *no*). We calculated the relative compliance across all questionnaires for each person so that a higher score indicated more compliance. When there were technical problems and participants had not received the AA questionnaire in time, they could not respond to the questionnaire. In these cases, we subtracted the number of AA questionnaires (which were missed due to technical problems) from the theoretical maximum number of completed AA questionnaires allowed by our protocol before we calculated the relative compliance.

#### Data analytic methods

To compare the experimental questionnaire length groups with respect to compliance and perceived burden, the analyses were the same as in Study 1. To compare the experimental groups with respect to within-person variability in mood and state extraversion and the relation between state extraversion and momentary mood, the within-person mood/extraversion scores were based on the items that were displayed in both groups (i.e., items that were displayed exclusively in the long questionnaire were excluded from all analyses). As in Study 1, H1 and H3 were corrected for multiple tests (Benjamini & Hochberg, 1995).

### Results

Table 5 presents the means, standard deviations, and within- and between-person correlations between the variables. For all momentary and daily measures, there was a substantial amount of within-person variance, ranging from 59% for daily perceived burden to 81% for state extraversion for the short questionnaire group and from 57% for daily perceived burden to 79% for state extraversion for the long questionnaire group.

**Table 5:** Descriptive statistics and bivariate correlations for the main variables presented separately for each experimental group (Study 2)

Group	Variable	1	2	3	4
Short questionnaire	1. Pleasant-unpleasant mood	—	.35***	– .10***	
	2. State extraversion	.42***	—	– .02	
	3. Daily perceived burden	– .30***	– .24**	—	
	4. Retrospective perceived burden	– .24**	– .19*	.83***	—
	*M*	4.90	3.13	2.40	2.71
	*SD*_*within*_	1.09	0.59	0.76	-
	*SD*_*between*_	0.79	0.29	0.63	0.85
	*N*_*persons*_	142	142	139	118
	*N*_*questionnaires*_^*a*^	4411	4411	1500	
Long questionnaire	1. Pleasant-unpleasant mood	—	.27***	– .09**	
	2. State extraversion	.38***	—	.05	
	3. Daily perceived burden	– .32***	– .06	—	
	4. Retrospective perceived burden	– .17	– .02	.81***	—
	*M*	5.01	3.12	2.51	2.77
	*SD*_*within*_	1.00	0.56	0.78	-
	*SD*_*between*_	0.72	0.31	0.71	0.92
	*N*_*persons*_	140	140	133	117
	*N*_*questionnaires*_^*a*^	4174	4174	1407	

*Note*. Between-person correlations are presented below the diagonal. Within-person correlations between the daily measures are presented above the diagonal. All *p* values are two-sided *p* values. For all daily measures, we extracted the mean (intercept) and standard deviation from the multilevel null model of the respective variable.

^a^*N* differed because the respective variables were assessed on different measurement occasions.

*p < .05. **p < .01. ***p < .001.

#### Perceived burden

Contrary to Hypothesis 1, daily perceived burden in the short questionnaire group (M = 2.40, SD = 0.67) was not lower than in the long questionnaire group (M = 2.51, SD = 0.77), *t*(268) = 1.29, one-tailed *p* = .099, 90% CI [− 0.03, 0.26], *d* = 0.14.

Retrospective burden was also not significantly lower in the short questionnaire group (M = 2.71, SD = 0.85) than in the long questionnaire group (M = 2.77, SD = 0.92), *t*(233) = 0.60, one-tailed *p* = .276, 90% CI [− 0.26, 0.12], *d* = 0.08.^9^

#### Compliance

Contrary to Hypothesis 2, the compliance rate in the short questionnaire group (M = .75, SD = 0.27) was not significantly higher than in the long questionnaire group (M = .72, SD = 0.28), *t*(219) = – 1.08, one-tailed *p* = .142, 90% CI [− 0.040, 0.008], *d* = –0.10.

#### Within-person variability

In line with Hypothesis 3, the short questionnaire group (estimate = 1.19, SE = 0.07) showed a higher degree of within-person variability in momentary pleasant-unpleasant mood than the long questionnaire group (estimate = 1.00, SE = 0.06), *z* = − 2.03, one-tailed *p* = .021, 90% CI [0.04, 0.35], *d* = − 0.24.^10^ The finding remained significant after we corrected the false discovery rate. Descriptively, the short questionnaire group (estimate = 0.35, SE = 0.02) also showed a higher degree of within-person variability in state extraversion than the long questionnaire group (estimate = 0.31, SE = 0.02), but this difference was not significantly different from zero, *z* = − 1.50, one-tailed *p* = .067, 90% CI [− 0.003, 0.076], *d* = − 0.13.^10^

#### Within-person relationships between time-varying variables

As can be seen in Table 6, the cross-level interaction between questionnaire length and state extraversion was significant, *t*(234) = − 2.98, one-tailed *p* = .003, 90% CI [− 0.29, − 0.08]. As expected, the slope coefficient for state extraversion was larger in the short questionnaire group (*b* = 0.65) than in the long questionnaire group (*b* = 0.47). As a quasi *R*^2^ measure of the cross-level interaction, we calculated the proportional reduction in the Level 2 variance of state extraversion slopes when questionnaire length was added as a predictor of the slopes (Raudenbush & Bryk, 2002). It was .04.

**Table 6:** Multilevel model (fixed effects) predicting momentary mood by state extraversion and questionnaire length (Study 2)

Model Predictor	Estimate	*SE*	*df*	*t*
Model 1:				
Intercept	4.92			
State extraversion	0.56	0.03	242.2	17.72***
Questionnaire length	0.16	0.09	268.2	1.67
Model 2:				
Intercept	4.90			
State extraversion	0.65	0.04	233.0	14.99***
Questionnaire length	0.20	0.09	268.6	2.01*
State Extraversion x Questionnaire Length	– 0.19	0.06	243.6	– 2.98**

*Note*. State extraversion was centered at the person mean. Questionnaire length was coded as 0 = short questionnaire group and 1= long questionnaire group.

*p < .05. **p < .01. ***p < .001.

#### Exploratory data analysis. *Predictors of compliance*

As in Study 1, none of the variables from the exploratory analyses were significantly related to compliance: gender, *t*(214) = 0.77, *p* = .440, 95% CI [− 0.02, 0.06]; age, *t*(217) = 0.05, *p* = .959, 95% CI [− 0.004, 0.004]; requesting personal feedback, *t*(220) = 0.31, *p* = .759, 95% CI [− 0.05, 0.07].

### Discussion

In Study 2, we experimentally manipulated another aspect of assessment intensity: questionnaire length. Unexpectedly, the questionnaire length groups did not differ in perceived burden (H1) or compliance (H2). In line with our expectations, the within-person variability in momentary mood (but not in extraversion) (H3) and the within-person relationship between state extraversion and momentary mood (H4) were smaller in the long (vs. short) questionnaire group.

Our results are in line with most previous nonexperimental research that found that questionnaire length was unrelated to perceived burden or compliance in an AA study (Jones et al., 2019; Ono et al., 2019; Soyster et al., 2019; Vachon et al., 2019). The only other experimental AA study that we know of that analyzed the effects of manipulated questionnaire length on burden and compliance (Eisele et al., 2020), however, found that longer questionnaires led to higher perceived burden and lower compliance. Whereas the number of items in the experimental groups were similar across studies (30 vs. 60 items per questionnaire in the study by Eisele et al., 2020, and 33 vs. 82 items per questionnaire in our study), the ways in which the greater number of items was achieved differed to some extent across studies: In the study by Eisele et al. (2020), most of the measured constructs were the same across groups, but the long questionnaire group had to answer two additional questions that referred to the pleasantness of the most important event and the stressfulness of situations since the last questionnaire. These additional questions might have caused participants in the long questionnaire group to think more about their daily negative experiences and therefore could have contributed to the effect that participants in the long (vs. short) questionnaire group perceived the study as more burdensome and showed a lower compliance rate.

Additionally, in the study by Eisele et al. (2020), participants needed to respond within a time frame of 90 s to an AA questionnaire (at random assessment times), whereas in our Study 2, participants had 45 min to respond to an AA questionnaire (at fixed assessment times). Therefore, participants in the study by Eisele et al. might have failed to respond to the questionnaire when they were in a situation that required their full attention (e.g., a conversation, cooking), whereas participants in our Study 2 had the option to simply delay their response by a few minutes in such a situation, thereby maintaining a higher compliance rate. As already discussed in Study 1, another possible explanation for not finding an effect of assessment intensity on compliance in our study is that the personal feedback incentive could have counteracted the decrease in compliance.

The finding that the degree of within-person variability in momentary mood and the within-person relation between state extraversion and mood was smaller in the long (vs. short) questionnaire group is in line with the notion that participants in the long questionnaire group responded in a more heuristic, less nuanced way to the repeated questionnaires (Fuller-Tyszkiewicz et al., 2013; Podsakoff et al., 2019). However, the effect on within-person variability was smaller for state extraversion, and the difference between groups was not significantly different from zero. One possible reason for this difference between momentary mood and state extraversion is that mood was assessed near (or at) the end of the AA questionnaire, whereas state extraversion was assessed at the beginning of the AA questionnaire. In line with this reasoning, research on positioning effects in cross-sectional surveys (Galesic & Bosnjak, 2009) found lower within-person variance in items that were assessed further away from the beginning of the questionnaire. Taken together, the effects of assessment intensity on burden, within-person variability, and the relation between two time-varying constructs were different between Study 1 (where sampling frequency was manipulated) and Study 2 (where questionnaire length was manipulated). We will come back to differences between these different types of manipulations of assessment intensity in the General discussion.

## General discussion

The aim of the current paper was to investigate whether differences in assessment intensity have an impact on the aspects of the data from an AA study. To address how assessment intensity was related to perceived burden, compliance, within-person variability, and the within-person relationship between time-varying variables, we used two different experimental manipulations of assessment intensity: sampling frequency (Study 1) and questionnaire length (Study 2). To our knowledge, the present research is the first to study within-person variability and the within-person relationship between time-varying variables as a function of experimentally manipulated assessment intensity in an AA study. Our main findings were that a higher sampling frequency affected only perceived burden but did not affect the other aspects of the AA data we investigated. A longer questionnaire, on the other hand, led to decreased intraindividual variability in momentary mood (but not in state extraversion) and a decreased within-person relationship between momentary mood and state extraversion, but it did not affect perceived burden or compliance.

With respect to compliance as the dependent variable, our experimental results are in line with a large body of previous research that found no impact of sampling frequency and questionnaire length on compliance (Conner & Reid, 2012; Jones et al., 2019; McCarthy et al., 2015; Ono et al., 2019; Soyster et al., 2019; Stone et al., 2003; Walsh & Brinker, 2016). One exception is Vachon et al.’s (2019) meta-analysis, which found that a higher sampling frequency but not questionnaire length led to a lower compliance. Similar to other studies, we had financially incentivized participants to reach certain levels of compliance in both the low and the high assessment intensity groups. This was done to ensure that the studies followed standard procedures, as financial incentives that are tied to compliance represent a very typical characteristic in AA studies (cf. Trull & Ebner-Priemer, 2020). Future experimental research might investigate the effects that remuneration schedules (e.g., the setting of different thresholds) have on compliance.

With respect to perceived burden (which had not been researched as extensively as compliance in previous research), we had expected that a higher assessment intensity would cause a higher burden, irrespective of the way the difference in objective time needed for study participation was realized (more questionnaires per day, as in Study 1, or more items per questionnaire, as in Study 2). However, as summarized above, these different manipulations of assessment intensity had different effects. One possible explanation for why we found an effect of sampling frequency but not questionnaire length on perceived burden is that participants may be more annoyed by more (vs. less) frequent interruptions in their daily lives than by a longer (vs. shorter) response duration (longer questionnaires). Note that for both questionnaire length groups in Study 2, the sampling frequency was the same as in the low sampling frequency group in Study 1 (i.e., three questionnaires per day). It is conceivable that an effect of questionnaire length on perceived burden shows up only when the study protocol requires a certain number of questionnaires per day (more than three). Given that the study by Eisele et al. (2020) is the only experimental AA study that had manipulated both factors simultaneously (but did not find an interaction between sampling frequency and questionnaire length), future research on the additive and potentially interactive effects of sampling frequency and questionnaire length on perceived burden is needed.

With respect to the degree of within-person variability and relation between time-varying constructs, our results indicated that a longer questionnaire length led to smaller values in these estimates but a higher sampling frequency did not. Note that the length of the questionnaire in Study 1 was similar to the length of the short questionnaire in Study 2 (i.e., around 30 items per questionnaire). That is, we cannot rule out the possibility that an effect of sampling frequency on within-person variability and the relation between time-varying constructs only shows up for longer questionnaires. Clearly, more research on the potential interaction between sampling frequency and questionnaire length on the degree of within-person variability and relation between time-varying constructs is needed. The psychological process behind the differential effect of sampling frequency versus questionnaire length on within-person variability and within-person relations might be that participants in an AA study with a high sampling frequency (but short questionnaires) might implicitly cope differently with the higher assessment intensity than participants in an AA study with many items per questionnaire (but low sampling frequency): If only relatively few items are assessed per measurement occasion, even with a high sampling frequency, participants might be able to produce high data quality (i.e., unbiased responses) by ignoring their perceived burden for the short duration of the questionnaire. On the other hand, long questionnaires might generally reduce participants’ effort in responding to the AA questionnaires (particularly at the end of the questionnaires; Galesic & Bosnjak, 2009), thereby reducing the quality of their data (i.e., producing biased responses). However, we can only speculate about how participants cope with the different types of demands an AA study poses. Note that Vachon et al. (2016) and Fuller-Tyszkiewicz et al. (2013) proposed an alternative explanation for a decrease in within-person variability over time. They suggested that over the course of an AA study, individuals’ accuracy in reporting momentary experiences increases due to the administration of repeated questionnaires, which might then lead to a reduction in random variance due to guessing. Further research is needed to replicate our findings and scrutinize the underlying processes.

## Limitations

Several limitations have to be considered when interpreting the results of our paper. First, both of our samples were from a student population. We do not know whether certain aspects of our findings depended on the sample characteristics. Therefore, our young, educated samples with large proportions of women restrict the extent to which our findings will generalize to another population. It is possible that the effects of sampling frequency or questionnaire length depend on age or sex, for example. However, we think that our findings should generalize to other student populations that are used in many other AA studies.

Second, it is likely that the motivation of the participants depended on the rewards that were given for participating in the study. For instance, in both of our studies, we offered participants the option to get personal feedback on their answers after the study had been completed. This might have increased participants’ motivation to provide more accurate responses or experience the study protocol as less burdensome for individuals who had more interest in the feedback. Additionally, these differences might have been influenced by personal characteristics. For example, participants with high neuroticism might be more interested in tracking momentary mood and state extraversion. Furthermore, we did not compensate the different groups with different rewards regardless of their assessment intensity. Especially in Study 2, the participants in the long questionnaire group were asked to invest more time and energy in participating than those in the short questionnaire group. If this difference between the groups resulted in less motivation to participate (e.g., because the participants thought the reward was not appropriate or they heard that another group in the same study had a shorter questionnaire), this could have resulted in reduced effort while responding to the AA questionnaires, which could have resulted in a reduction in the within-person variability as well as in the relations between the time-varying constructs. However, we do not know if participants’ motivation depended on the rewards that were given. Future research should investigate the effects of rewards on data quantity and quality.

Third, we had limited opportunities to control for careless responders in our sample. We administered one self-reported single item that could indicate invalid responders and a consistency index that could indicate whether some questionnaires were answered inconsistently. If careless responding was not sufficiently controlled for, it could bias the results of our investigation. Furthermore, there might be different types of careless responders in AA studies. Some careless responders might increase the within-person variability, whereas others might decrease the within-person variability. To our knowledge, there are no guidelines for how to deal with careless responders in AA. Future studies should identify the careless responding indices (Meade & Craig, 2012) that are suitable for use in AA studies, identify possible types of careless responders, and establish guidelines for how to deal with careless responders.

Finally, we manipulated only two central aspects of the design in an AA study. However, this leaves many other potential aspects (e.g., study duration, distribution of assessments across the day, type of sampling [time-, interval-, or event-contingent sampling], financial compensation, content of the questions, item difficulty, order of the measured items, and the instructions or the software used to signal the participants) that might have effects on the quantity or quality of AA data. Furthermore, we do not know whether our results can be generalized to other (e.g., higher) sampling densities and other (e.g., longer) questionnaire lengths.

## Conclusions

The present research is the first to experimentally manipulate assessment intensity to investigate changes in within-person variability and the within-person relationship between time-varying variables. Furthermore, we found that a higher assessment intensity can affect within-person variability and relations between time-varying constructs without increasing participants’ perceived burden. Although further validation of the findings is essential, we hope that future researchers will integrate our findings when planning an AA study.

## Data Availability

The data sets generated during the current studies and the respective codes are available on the OSF repository (Study 1: https://osf.io/vw3gf/?view_only=b6f9f08a6b5941eb9c17a4951d1d0cd2; Study 2: https://osf.io/xt3rf/?view_only=6e6bf509c6374759b56faac680c55825). Studies 1 and 2 were preregistered (see respective URLs).

## Footnotes

1 Study 1 was preregistered on February 1, 2019 with all hypotheses and methods of data analysis. The hypotheses for Study 2 were preregistered in January 2020. Please note that the preregistration documents include hypotheses that were not tested/reported in the present paper. The reason is that testing/reporting all hypotheses would have gone beyond the scope of a single paper. The preregistered hypotheses that were not investigated in the current research will be tested and reported in separate papers. Also note that the test of Hypothesis 3 (on within-person variability) was preregistered for momentary mood as the variable of interest but not for state extraversion. When analyzing the data, we realized that an important piece of information would be missing if we did not test and report the effects on the degree of within-person variability in state extraversion, too. The preregistrations can be found on the OSF pages of the respective studies (Study 1: https://osf.io/vw3gf/?view_only=b6f9f08a6b5941eb9c17a4951d1d0cd2; Study 2: https://osf.io/xt3rf/?view_only=6e6bf509c6374759b56faac680c55825).

2 To define an inconsistency index (Meade & Craig, 2012) for each measurement occasion in an AA study, items that are extremely similar in content and demonstrate a very large (negative or positive) within-person correlation are needed. In our study, bipolar momentary mood items (e.g., for the subscale pleasant-unpleasant mood: good-bad vs. happy-unhappy vs. unpleased-pleased vs. unwell-well; within-person intercorrelations across all subscales ranged from r = |.55| to |.73|) met these criteria. We defined inconsistent responding at a particular measurement occasion as illogical responses across mood item pairs with responses near (or at) the extremes of the scale (Categories 1 or 2 vs. 6 or 7). For example, response patterns, such as feeling “very happy” and “very unwell” at the same time or feeling “very happy” and “very bad” at the same time were categorized as inconsistent responses. More information about the momentary mood items can be found on the OSF page of Study 1 (https://osf.io/vw3gf/?view_only=b6f9f08a6b5941eb9c17a4951d1d0cd2).

3 Only the relevant scales for the analyses used in this investigation are described. An overview of all measured constructs can be found on the OSF page of this project (https://osf.io/vw3gf/?view_only=b6f9f08a6b5941eb9c17a4951d1d0cd2).

4 Note that independent t tests (and not multilevel regression analyses) had been preregistered for testing Hypothesis 1 (on daily perceived burden) and Hypothesis 2 (on compliance). However, for reasons of consistency and to avoid the need to aggregate values by hand, we switched to multilevel analyses during the peer review process. To remain close to the preregistered data analytic method tests, we additionally report means and Cohen’s d values along with the results of the multilevel regressions.

5 We performed additional exploratory analyses on the linear effect of the day of the study on daily perceived burden, with the day of the study centered on the 4th day and investigated the interaction between time (the day of the study) and sampling frequency. We conducted the data analytic steps correspondingly for Hypothesis 4. In the final model (with the interaction term), the main effect of the day of the study was significant, t(239) = 3.69, p < .001, 95% CI [0.03, 0.09]. The cross-level interaction between sampling frequency and the day of the study was significant, t(240) = − -3.52, p < .001, 95% CI [− −0.12, − −0.04], which means that the low sampling frequency group (b = 0.06) had a larger regression coefficient than the high sampling frequency group (b = − -0.02).

6 To estimate the effect size of this test, we person-centered momentary mood or state extraversion and aggregated the variances for each person to get a value for the within-person variance. This allowed us to estimate Cohen’s d for the effect size of this analysis.

7 We defined inconsistent responding at a particular measurement occasion in the same way as we did in Study 1. However, in Study 2, there were only two momentary mood items (within the same subscale) that were presented to both experimental groups (short vs. long questionnaire), and hence, the inconsistency index was based on these two items from the momentary pleasant-unpleasant mood subscale (the within-person intercorrelation was r = − -.73). More information about the momentary mood items can be found on the OSF page of Study 2 (https://osf.io/xt3rf/?view_only=6e6bf509c6374759b56faac680c55825).

8 Only the relevant scales for the analyses used in this investigation are described. An overview of all measured constructs can be found on the OSF page of this project (https://osf.io/xt3rf/?view_only=6e6bf509c6374759b56faac680c55825).

9 We conducted additional exploratory analyses on the linear effect of the day of the study on daily perceived burden, with the day of the study centered on the midpoint of the assessment duration (7.5) and investigated the interaction between time (the day of the study) and questionnaire length. We conducted the data analytic steps correspondingly for Hypothesis 4. In the final model (with the interaction term), the main effect of the day of the study was significant, t(238) = 6.44, p < .001, 95% CI [0.03, 0.06]. The cross-level interaction between questionnaire length and the day of the study was significant, t(241) = − -2.57, p = .011, 95% CI [− 0.04, − 0.01], which means that the short questionnaire group (b = 0.04) had a larger regression coefficient than the long questionnaire group (b = 0.02).

10 To estimate the effect size of this test, we person-centered momentary mood or state extraversion and aggregated the variances for each person to get a value for the within-person variance. This allowed us to estimate Cohen’s d for the effect size of this analysis.
